# Management of Retinopathy of Prematurity in the United Arab Emirates: A Consensus Statement From the Emirates Society of Ophthalmology

**DOI:** 10.7759/cureus.103096

**Published:** 2026-02-06

**Authors:** Abeer K Al Ali, Noura Al Qassimi, Hanan Al Shamsi, Syed Muhammad Asad Ali, Tulika Kar MS, Laila Obaid, Firdaus Sukhi, Hassan Salim Al Hasid, Ayesha Khan, Muhammad Irfan Khan, Darakhshanda Khurram

**Affiliations:** 1 Department of Ophthalmology, Burjeel Medical City, Abu Dhabi, ARE; 2 Department of Ophthalmology, Sheikh Khalifa Medical City, Abu Dhabi, ARE; 3 Department of Ophthalmology, Canadian Care Medical Center, Al Ain, ARE; 4 Department of Ophthalmology, Moorfields Eye Hospital, Abu Dhabi, ARE; 5 Department of Pediatric Ophthalmology, Al Fujairah Hospital, Emirates Health Services, Fujairah, ARE; 6 Department of Neonatology and Pediatrics, SEHA - Corniche Hospital, Abu Dhabi, ARE; 7 Department of Ophthalmology, Sheikh Khalifa Medical City, Ajman, Ajman, ARE; 8 Department of Ophthalmology, Dubai Hospital, Dubai, ARE; 9 Department of Ophthalmology, Al-Shifa Trust Eye Hospital, Rawalpindi, PAK; 10 Department of Pediatric Ophthalmology, Sheikh Khalifa Medical City, Abu Dhabi, ARE

**Keywords:** premature newborns, retinopathy of prematurity, screening protocols, treatment modalities, united arab emirates

## Abstract

Retinopathy of prematurity (ROP) is a leading cause of preventable childhood blindness worldwide and affects a substantial proportion of very preterm or very-low-birth-weight infants. In the United Arab Emirates (UAE), hospital-based cohorts report a notable incidence of ROP among preterm infants, underscoring the need for standardized protocols.

This Nominal Group Technique (NGT)-based national expert consensus aims to provide unified guidance for early detection, effective management, and improved outcomes for infants at risk of ROP in the UAE. A nationally representative panel of UAE-based consultant ophthalmologists, with neonatologists as non-voting advisors, developed recommendations for the International Classification of Retinopathy of Prematurity, Third Edition (ICROP-III) classification, screening, and management. Key management strategies include timely laser photocoagulation for treatment-requiring ROP, where feasible, and intravitreal anti-vascular endothelial growth factor (VEGF) therapy as a preferred or initial option in posterior disease and aggressive ROP (A-ROP), with prolonged surveillance for reactivation.

Unmet needs include system-level gaps (standardized documentation, integrated tracking, and follow-up pathways) and educational gaps, particularly neonatologist and neonatal intensive care unit (NICU) staff engagement in prevention and long-term follow-up.

## Introduction and background

Retinopathy of prematurity (ROP) is a sight-threatening disease and a leading cause of childhood blindness worldwide, primarily affecting preterm infants [1]. Since its initial description in the early 1940s, the incidence of ROP has followed three epidemic phases globally, reflecting evolving patterns of neonatal survival and care [2]. Advances in neonatal intensive care over recent decades have substantially improved survival rates among preterm infants; however, these improvements have been accompanied by an increased incidence of ROP, particularly in regions where advanced neonatal services have become more widely accessible [3]. Population-based studies report that ROP occurs in approximately 12.6% to 34.1% of infants born at a gestational age (GA) of <30-32 weeks, those with very low birth weight (VLBW; <1,500 g), or those with an unstable clinical course [2,4,5].

From a pathological perspective, ROP is a vasoproliferative retinal disorder characterized by a biphasic process. An initial phase of impaired or arrested retinal vascular growth occurs following premature exposure to relative hyperoxia, followed by a second phase in which hypoxia in the avascular retina stimulates abnormal neovascularization. If untreated, this process may progress to fibrovascular proliferation, retinal detachment, and irreversible vision loss [6].

As a result, ROP screening has become an essential component of neonatal care for at-risk infants [7]. Timely diagnosis and appropriate intervention - most commonly laser photocoagulation or intravitreal anti-vascular endothelial growth factor (VEGF) therapy - have been shown to significantly reduce disease progression and improve visual outcomes [8]. Despite these advances, ROP management remains challenging due to its multifactorial etiology, involving oxygen exposure, systemic comorbidities, genetic susceptibility, and postnatal growth factors [9]. Moreover, there is no universally adopted “gold-standard” screening protocol, with notable differences between major international guidelines in screening thresholds, timing, and follow-up schedules, reflecting geographic- and population-specific variability in ROP risk [10].

In the United Arab Emirates (UAE), previous reports have documented an ROP incidence of approximately 27% among preterm infants, highlighting a substantial national disease burden [11]. Although neonatal care in the UAE has advanced considerably, the absence of unified, nationally adapted protocols has resulted in variability in screening and management practices. Therefore, this consensus statement was developed to provide standardized, evidence-informed recommendations for ROP classification, screening, and treatment. The consensus reflects the collective input of a nationally representative panel of UAE-based ophthalmology experts, with neonatologists contributing contextual clinical perspectives and aims to support early detection, consistent management, and improved outcomes for infants at risk of ROP.

## Review

Methodology

Study Design and Panel Recruitment

This national consensus adopted the Nominal Group Technique (NGT) to develop recommendations for the screening and management of ROP. In brief, the process comprised structured idea generation, moderated discussion, and iterative voting rounds, with a predefined consensus threshold. The Emirates Society of Ophthalmology invited a predefined panel of 10 experts, aligned with methodological guidance for NGT recommending approximately 7-12 participants, to ensure adequate diversity and geographic representation while maintaining feasibility and effective group interaction. The panel consisted of consultant-level, licensed ophthalmologists actively practicing in the UAE, representing both public and private sectors and different UAE regions. Eligibility criteria included: (1) a primary clinical focus in pediatric ophthalmology and/or retinal disease, (2) ≥5 years of post-training experience, and (3) direct involvement in ROP screening, diagnosis, or treatment within the preceding two years. Subspecialty expertise and institutional practice context were considered to ensure clinical relevance; accordingly, panel members were affiliated with academic institutions and tertiary referral centers, including high-volume neonatal intensive care units (NICUs) managing ROP.

Experts were either active members of the Emirates Society of Ophthalmology or formally nominated by the Society, and all participants were required to commit to all predefined steps of the consensus process. Exclusion criteria included lack of active ophthalmic practice in the UAE, absence of recent hands-on involvement in ROP management, or failure to complete key consensus steps (e.g., non-response to one or more voting rounds, or absence from the final ratification meeting).

Conflict of interest (COI) disclosure and management: To enhance transparency and reproducibility, all voting panel members completed written COI disclosure forms prior to participation. Disclosures were reviewed by the organizing committee, and any potential conflicts were openly acknowledged and managed throughout the consensus process. Individuals with unresolved conflicts, likely to bias judgment (e.g., undisclosed financial relationships directly related to ROP therapeutics or devices), were excluded.

Multidisciplinary participation: In addition to ophthalmologists, neonatologists with ≥3 years of experience in ROP programs were invited as non-voting advisors. They contributed actively during discussions by providing contextual input on neonatal care pathways, the feasibility of screening schedules, and practical implementation considerations. Their input informed the refinement and clinical applicability of recommendations, while voting remained restricted to the ophthalmology expert panel.

This consensus process did not involve patient recruitment or identifiable patient data; therefore, inclusion and exclusion criteria applied solely to expert participants. The activity was deemed exempt from formal ethical review, in accordance with applicable national research regulations and institutional governance frameworks for expert consensus and guideline development.

Literature Review and Pre-meeting Questionnaire

The literature component was conducted as a targeted narrative evidence review to inform questionnaire development and support consensus discussions; it was not designed as a systematic review or meta-analysis. Prior to the consensus meeting, participants received a structured pre-meeting questionnaire designed to elicit baseline perspectives and identify priority issues for discussion. The questionnaire comprised thematic domains covering ROP classification, screening criteria, treatment modalities, and management of complications, and included open-ended questions to capture clinical rationale and supporting evidence. For reproducibility, the questionnaire content is summarized by the number of major domains and questions (Appendix A).

To inform questionnaire development and evidence summaries, an online bibliographic search was conducted in MEDLINE (via PubMed) from database inception to August 2023, limited to English-language publications. Reference lists of key articles were manually screened to ensure comprehensive capture of relevant evidence. A standardized search strategy was used, with iterative refinement of Boolean operators to optimize sensitivity and specificity across related query iterations. The search terms included: (("Retinopathy of Prematurity"[MeSH] OR retinopathy of prematurity OR ROP) AND ("Consensus"[MeSH] OR consensus statement OR "Practice Guidelines as Topic"[MeSH] OR clinical guidelines OR best practices) AND ("Disease Management"[MeSH] OR disease management OR healthcare management)). Eligible sources included clinical guidelines, consensus statements, randomized trials, and observational studies relevant to ROP classification, screening, treatment, and follow-up. Evidence was prioritized by methodological quality and relevance to the UAE context. Exclusion criteria included non-English publications, non-human studies, and articles not addressing ROP screening/management outcomes relevant to the consensus questions.

Evidence quality informed both question prioritization and the strength of recommendations. Questions and draft statements were primarily derived from higher-quality evidence (Level 1), classified per Wright et al. [12]. Lower-quality evidence was incorporated only when (i) high-level evidence was unavailable, (ii) region-specific considerations required local contextualization, or (iii) expert guidance was needed to address clinically significant evidence gaps.

Evidence was summarized qualitatively (narrative synthesis). When multiple studies reported comparable outcomes (e.g., incidence, treatment rates), results were reported as ranges and interpreted in light of the study setting, population, and design. Higher-level evidence was weighted more heavily during statement drafting and voting.

Because this was not a systematic review, dual independent screening, a PRISMA flow diagram, and formal risk-of-bias scoring were not performed. No meta-analysis or quantitative pooling was undertaken; descriptive estimates are reported from the cited literature, as ranges, to support the consensus context.

NGT Consensus Meeting, Voting, and Definition of Consensus

The consensus process comprised two formal NGT rounds. During the meeting, proposed statements were discussed in a structured, moderated format and subsequently subjected to voting. Consensus was defined a priori as ≥80% agreement among voting panel members, consistent with thresholds commonly used in NGT guideline development. Statements not meeting the consensus threshold were revised, re-discussed, and subjected to subsequent voting. Where appropriate, anonymous voting was used to minimize dominance bias and ensure equitable contribution.

Recommendations were categorized as evidence-based, expert-consensus-based, or hybrid, depending on the strength and consistency of available evidence across domains. In addition, alignment with existing international guidance (e.g., AAP, UK ROP, and ROPNet) was reviewed, with clearly justified adaptations made to reflect local neonatal demographics, healthcare infrastructure, and disease burden.

Results and discussion

Overview of the Incidence, Risk Factors, and Pathogenesis of ROP

The recommendations presented in this consensus represent a hybrid approach, integrating evidence from published literature with structured expert opinion and are largely aligned with established international guidelines (including AAP and UK ROP guidelines), while being adapted to the local epidemiology and healthcare context of the UAE.

Incidence and natural history of ROP: According to the World Health Organization (WHO), approximately 13.4 million infants were born preterm (defined as GA <37 weeks), and 23.4 million were small for gestational age (SGA) globally in 2020 [13]. Infants who are SGA represent a population with impaired fetal growth and higher vulnerability to postnatal complications, including ROP. Advances in neonatal care have markedly improved survival among extremely preterm infants; however, this success has been accompanied by a rising incidence of ROP. A previous meta-analysis estimated that approximately 21.7% of preterm infants developed ROP, encompassing all disease stages [14].

The incidence of ROP varies substantially across regions. Data from the United States indicate that up to 68% of VLBW infants develop ROP, with 36.9% exhibiting clinically significant (pre-threshold) disease [15]. In Europe, reported ROP incidence ranges from 12.6% to 21.9% among preterm infants [4,16], reported as ranges across individual studies rather than pooled estimates. These variations largely reflect differences in neonatal care practices, oxygen management, nutrition, and screening protocols [17]. Differences between studies likely reflect heterogeneity in GA/BW (birth weight) distributions, oxygen practices, screening thresholds, and care settings.

In the Middle East, reported ROP incidence ranges from 11% to 40.4% among preterm or VLBW infants [18,19]. In the UAE, hospital-based cohort studies have reported an ROP incidence of approximately 27% in preterm infants, highlighting the significant national burden of disease and the need for standardized management strategies [11].

Risk factors of ROP: The most important risk factors for ROP are the degree of prematurity and low BW. Infants born at a GA below 28 weeks or with a BW under 1,000 g are at particularly high risk for severe disease [20]. The incidence and severity of ROP are inversely related to both GA and BW, as demonstrated by the CRYO-ROP study, which showed a 27% reduction in threshold ROP risk for every 100 g increase in BW, and a 19% reduction for each additional week of gestation [21,22].

Neonatal factors, particularly oxygen exposure, play a central role in ROP pathogenesis. While supplemental oxygen is essential for survival, prolonged or excessive exposure can disrupt normal retinal vascular development [23]. Higher oxygen concentrations, longer duration of oxygen therapy, and mechanical ventilation have all been associated with increased ROP risk [24]. Clinical trials evaluating optimal oxygen saturation targets have yielded conflicting results. While lower saturation targets may reduce ROP incidence, they have been associated with increased mortality in some studies, underscoring the need for balanced oxygen targeting in current NICU practice [25-28]. 

Although randomized trials evaluating oxygen saturation targets in preterm infants have reported conflicting results with respect to ROP incidence and mortality, their collective findings highlight the need for a balanced and individualized approach in NICU practice. Lower oxygen saturation targets may reduce the risk of severe ROP, but have been associated with increased mortality in some studies, whereas higher targets may improve survival at the expense of increased ROP risk. Consequently, current NICU management should prioritize careful oxygen titration within unit-specific protocols, continuous monitoring, and close interdisciplinary collaboration between neonatologists and ophthalmologists. Emphasis should be placed on avoiding both hypoxia and hyperoxia, recognizing that stable oxygen control over time - rather than rigid adherence to a single saturation threshold - is critical for optimizing overall neonatal outcomes.

Maternal and perinatal factors, including preeclampsia, maternal diabetes, advanced maternal age, intrauterine growth restriction, multiple gestations, late-onset sepsis, and low Apgar scores, have also been associated with ROP risk [20,26]. Postnatal factors, such as bronchopulmonary dysplasia, respiratory distress syndrome, apnea of prematurity, prolonged mechanical ventilation, anemia requiring transfusion, necrotizing enterocolitis, and intraventricular hemorrhage, further increase susceptibility [11,26,29]. Conversely, breastfeeding and adequate postnatal nutrition have been shown to reduce ROP risk [30,31].

From a mechanistic perspective, insulin-like growth factor-1 (IGF-1) is critical for normal retinal vascular development. Premature infants experience a postnatal decline in IGF-1 levels, compared with in utero concentrations, contributing to delayed vascular growth and increased ROP susceptibility [32,33]. As IGF-1 levels later rise, excessive VEGF activity may drive pathologic neovascularization [34]. This understanding supports the importance of optimizing postnatal growth and nutrition, and may inform future preventive or therapeutic strategies.

Emerging evidence suggests a genetic contribution to ROP susceptibility, particularly involving polymorphisms in angiogenesis-related genes such as VEGF. At present, however, these findings remain largely investigational, and are not yet incorporated into routine clinical risk stratification or management [35]. Pathogenesis of ROP: VEGF plays a central role in normal retinal vascular development; therefore, disturbances in oxygen balance can adversely affect retinal vascularization [36,37]. The pathogenesis of ROP is classically described as a two-phase process, consisting of an initial phase of delayed vascular growth (vaso-attenuation), followed by a second phase of pathological neovascularization (vaso-proliferation).

Shortly after premature birth, the vaso-attenuation phase is characterized by arrest of normal retinal vascular growth due to disruption in the supply of growth factors. This is compounded by the abrupt transition from the relatively hypoxic intrauterine environment to higher postnatal oxygen levels. Hyperoxia suppresses VEGF expression, further inhibiting physiologic retinal vascularization, and leading to areas of avascular retina [38,39]. This mechanism underscores the importance of careful oxygen regulation in NICU practice.

In the second phase, the persistently avascular peripheral retina becomes hypoxic, stimulating upregulation of angiogenic factors, particularly VEGF. While VEGF is essential for vascular growth, excessive expression in this context results in abnormal neovascularization. Newly formed vessels proliferate toward the vitreous rather than along the retinal plane, rendering them fragile and prone to hemorrhage, fibrosis, and tractional retinal detachment [36]. This pathological sequence provides the biological rationale for laser photocoagulation, which ablates avascular retina, and for anti-VEGF therapy, which suppresses abnormal angiogenesis.

This mechanistic understanding underpins current preventive strategies focused on careful oxygen regulation and optimized postnatal growth, and it also provides a biological rationale for targeted therapies, such as laser photocoagulation and anti-VEGF agents, while informing the development of future interventions aimed at modulating retinal angiogenesis.

International Classification of Retinopathy of Prematurity, Third Edition (ICROP-III) classification of ROP: Regardless of the imaging modality used, ROP should be classified according to the ICROP-III, introduced in 2021, which provides a standardized framework for describing disease location, severity, and activity [40]. ICROP-III was developed to address limitations of ICROP-II and to accommodate evolving treatment paradigms, particularly the widespread use of anti-VEGF therapy, and challenges related to disease regression and reactivation. In addition to the traditional five parameters (zone, stage, plus disease, extent, and presence of aggressive ROP (A-ROP)), ICROP-III formally incorporates the terms regression and reactivation [40].

A central component of ICROP-III is zonal classification. Zone I encompasses a central retinal area extending twice the distance from the optic disc to the foveal center, and can be assessed clinically using a 28-diopter lens [41]. Zone II extends from the edge of Zone I to the nasal ora serrata, while the remaining temporal crescent constitutes Zone III [40]. Zone III classification requires complete nasal vascularization to the ora serrata, without ROP in the two most nasal clock hours. ICROP-III also introduces the concept of a “notch,” defined as a posterior extension of ROP by one to two clock hours, which may influence disease staging and treatment decisions [40].

Disease severity is classified into Stages 1-3 (vascular changes) and Stages 4-5 (fibrovascular proliferation and retinal detachment). In ICROP-III, Stage 5 is further subdivided into three categories, reflecting varying anatomical configurations, with important prognostic and surgical implications [40]. Clinically, severity is determined by the most advanced stage present in the retina.

Plus disease, defined by dilation and tortuosity of posterior retinal vessels, may occur at any stage and signals the need for urgent intervention. Pre-plus disease describes vascular changes insufficient to meet plus disease criteria. ICROP-III emphasizes a comprehensive assessment of posterior pole vessels, particularly within Zone I [40]. Disease extent is documented in clock hours (1-12), allowing standardized quantification of circumferential involvement.

Finally, ICROP-III replaces the term aggressive posterior ROP (AP-ROP) with A-ROP, reflecting recognition that aggressive disease is not limited to posterior locations. A-ROP is characterized by rapid progression and severe vascular abnormalities, and is associated with a poor prognosis if not treated promptly, reinforcing the need for early recognition and urgent management [40,42].

Screening Protocols

Timely screening and intervention are essential to prevent severe visual impairment due to ROP. Effective screening requires accurate identification of high-risk infants, appropriate timing of the initial examination, and structured follow-up protocols [3]. Although international screening guidelines exist, significant variability remains due to differences in neonatal demographics, healthcare infrastructure, and available resources [10], necessitating UAE-specific adaptation of screening practices.

Internationally, American guidelines recommend screening infants born at <31 weeks’ GA or with BW <1501 g, as well as selected larger or more mature infants with unstable clinical courses [43]. UK guidelines recommend screening infants born at <32 weeks’ GA or BW <1501 g [7]. The recommendations presented here are consensus-based, aligned with international guidance, and adapted to local UAE practice patterns and infrastructure.

In the UAE, ROP screening is recommended for infants born at GA <32 weeks or BW <1501 g. Initial screening should occur at 31-32 weeks’ postmenstrual age for infants born before 27 weeks’ GA. For infants born between 27 and 32 weeks’ GA, and for infants >32 weeks’ GA with BW <1501 g, initial screening should be performed at four to five weeks’ postnatal age (Table 1). Infants with unstable clinical courses should be screened even if standard criteria are not met. In summary, screening timing is determined by gestational maturity at birth, with earlier screening for the most premature infants.

**Table 1:** Recommended Screening Protocol in United Arab Emirates Abbreviations: GA: gestational age; ROP: retinopathy of prematurity; PMA: postmenstrual age

Item	Recommendations
Who Should Be Screened?	All infants born at <32 weeks’ gestational age (up to 31 weeks) or with birth weight ≤1500 g should be screened for ROP. Meeting either criterion is sufficient for inclusion.
Initial Screening	GA <27 Weeks: At 30-31 weeks postmenstrual age. GA 27-32 Weeks: At 4-5 weeks postnatal age. GA >32 Weeks and BW <1501 g: At 4-5 weeks postnatal age.
Frequency of Screening	Weekly: If vessels end in zone I or posterior zone II, plus or pre-plus disease is present, or stage 3 ROP is identified in any zone. Biweekly: All other cases.
Screening Termination	Stop when: Retina fully vascularized to ora serrata (360°), Vascularization reaches zone III with no prior ROP in zones I/II, PMA 45 weeks with no type 1/aggressive ROP, - ROP regressed in zone III with no abnormal tissue. If ROP present: Stop if disease is stable or resolving (ridge turns white, vessels cross demarcation, lesions replaced by scar tissue).

Follow-up frequency depends on retinal findings. Weekly examinations are indicated if vessels end in Zone I or posterior Zone II, if plus or pre-plus disease is present, or if Stage 3 ROP is identified in any zone [7]. Biweekly examinations are appropriate for all other cases. Alternative follow-up schedules, based on zone and stage, have been described in the American Academy of Pediatrics guidelines [43].

ROP examinations can be stressful for infants and families. Neonatologists and NICU nursing staff play a critical role in ensuring physiological stability, parental counseling, and safe examination conditions. Parents should receive oral and written information prior to screening, and any postponed examination should be rescheduled within one week. Mydriatic agents should be administered cautiously, using the lowest effective dose, with monitoring for systemic side effects, such as tachycardia or hypertension [44]. Comfort measures, including oral sucrose, nesting, and swaddling, are recommended [45].

Screening examinations should be performed by an ophthalmologist trained and experienced in ROP screening. Binocular indirect ophthalmoscopy using 20D, 28D, or 30D lenses remains the standard method, with scleral depression employed as needed to visualize the peripheral retina [46]. Wide-field retinal imaging systems may complement examinations, facilitate telemedicine, and provide standardized documentation. When imaging is used, infants with methicillin-resistant *Staphylococcus aureus* (MRSA) should be examined last, in accordance with institutional infection control practices [47]. Documentation should follow ICROP-III terminology to ensure consistency.

Screening may be discontinued when the retina is fully vascularized to the ora serrata, vascularization reaches Zone III without prior Zone I or II disease, or when infants reach 45 weeks’ postmenstrual age without Type 1 or A-ROP [48]. Continued surveillance is recommended in infants <37 weeks’ GA until stability or regression is confirmed, particularly in those with prior ROP [49].

After discharge, all preterm infants should undergo visual assessments during the first year of life. Infants treated for ROP require longer-term follow-up into preschool age to monitor visual development, refractive error, and strabismus [48]. Infants treated with anti-VEGF require closer and prolonged follow-up due to the risk of late reactivation and persistent avascular retina (PAR) [50]. Early referral pathways and access to visual rehabilitation services are essential for infants with ROP-related complications.

Management of ROP

Indications for ROP treatment: ROP may progress to advanced stages or spontaneously regress. Most preterm infants with ROP experience spontaneous regression and do not develop severe disease. In a previous report, the incidence of severe ROP (Stages 4-5) was 0.2%, compared with 9.5%-11.6% for Stages 1-3; the overall incidence of ROP requiring treatment was 5.7% [51]. Other reports estimated that 1.2%-6.8% of infants require treatment [5,52]. These rates reflect prior reports and may vary across settings depending on contemporary neonatal practice, case mix, and local screening thresholds, particularly by GA, BW, and systemic illness severity [5,51,52]. Infants with VLBW, extremely low BW, or lower GA have a higher risk of severe (“threshold”) ROP [2].

International guidance generally recommends treatment for threshold ROP, early treatment for ROP (ETROP) Type 1 (treatment-requiring) pre-threshold ROP, A-ROP, and persistent active disease beyond a corrected age of 45 weeks, whereas ETROP Type 2 disease is typically managed with close observation and timely follow-up [53]. In the UAE, we recommend ROP treatment for the following indications [53]: Zone I: any stage with plus disease; Zone I: Stage 3 without plus disease; Zone II: Stage 2 with plus disease; Zone II: Stage 3 with plus disease.

These indications are consistent with ETROP Type 1 (treatment-requiring) disease categories [53]. Once the decision to treat is made, treatment should be initiated within 48-72 hours (two to three days) to reduce the risk of rapid progression, consistent with guideline-based recommendations and clinical practice standards referenced in the manuscript [53].

In NICU settings, ROP treatment decisions should be accompanied by documented informed consent from a parent or legal guardian. Clinicians should explain the diagnosis and severity, treatment options (including expected benefits and risks), alternatives (including observation, when appropriate), and the planned follow-up schedule. For intravitreal anti-VEGF therapy, consent discussions should additionally address the need for extended follow-up for reactivation, and the rationale for agent selection and dosing, as used in the local protocol [53]. Consent should be documented in the medical record, and communication should be provided in clear language appropriate to the family’s needs, in coordination with the NICU team. Additional ethical considerations specific to off-label anti-VEGF use and extended surveillance requirements are detailed in the Anti-VEGF Therapy for ROP section.

Laser treatment for ROP: Laser photocoagulation is a standard treatment modality for ROP and is widely used to ablate avascular peripheral retina, thereby reducing vasoproliferative signaling and lowering the risk of progression to retinal detachment [54,55]. Several studies have reported high structural success rates (approximately 92%-95%), although outcomes may vary by GA, BW, disease zone (posterior vs peripheral), and severity at the time of treatment [56]. Compared with cryotherapy, laser treatment is associated with fewer complications and improved structural and functional outcomes, supporting its role in contemporary clinical practice [57]. Laser efficacy may be reduced in the setting of delayed diagnosis or advanced disease [58].

In our practice, we recommend laser therapy for Zone II ROP with Stages 2-3 and plus disease. Depending on institutional protocol, laser photocoagulation may be performed with sedation, under general or local anesthesia. With intravenous fluids and continuous cardiorespiratory monitoring, oral feeding should be discontinued three hours before the procedure. Pupil dilation may be achieved using cyclopentolate 0.5% and phenylephrine 2.5% eye drops; tropicamide 0.5% is preferable to tropicamide 1%, due to safety considerations in neonates [59]. Key laser parameters, peri-procedural practices, and follow-up recommendations are summarized in Table 2.

**Table 2:** Treatment Recommendations for ROP in the United Arab Emirates Abbreviations: VEGF: vascular endothelial growth factor; ROP: retinopathy of prematurity; NICU: neonatal intensive care unit

Item	Laser Therapy	Anti-VEGF
Indications	Zone II stage 2 with plus disease; Zone II stage 3 with plus disease	A-ROP; Zone I (any stage, per local protocol); Zone II stage 3 without plus disease; Zone II stage 2 without plus disease
Specifications/Dose	Power: 150-250 mW (may be increased if no response). Duration: 200 ms. Interval: 150-200 ms. Apply confluent burns, spaced at 0.5-1 burn widths, methodically covering the avascular retina.	Bevacizumab 0.625 mg/0.025 mL; Ranibizumab 0.25 mg/0.025 mL
Follow-Up Schedule	Post-procedure check typically at 1 week, then weekly for 1 month to monitor recurrence. Treat any untreated areas (particularly near the vascular-avascular junction) with additional laser.	-
Special Considerations	Laser should be performed by personnel experienced with neonatal laser protocols. Demarcate the treatment area and use manufacturer-recommended laser safety goggles. A 20-D lens may be used for posterior pole orientation/demarcation, followed by a 28-D or 30-D lens to visualize and treat the periphery. Consider post-laser imaging to identify gaps. In general anesthesia, an endotracheal tube may be preferred over a laryngeal mask.	Use a different sterile surgical set for each eye. Prepare each eye separately with povidone-iodine (per institutional protocol). Injection location: 1.7-2.0 mm posterior to the limbus. Prefer a negative-pressure room when available; if transfer is unsafe, bedside injection may be performed (preferably in the incubator). Treat each eye as a separate procedure with appropriate monitoring; a nurse and respiratory therapist should be present. Pre-procedure analgesia (e.g., morphine or ketamine) may be administered per NICU/pediatric guidance.
ROP Recurrence After Anti-VEGF	Laser is indicated 6-8 weeks after injection and is strongly recommended for persistent avascular retina, particularly when it exceeds 2 disc diameters.	-

Because Zone I disease and A-ROP are posterior, rapidly progressive phenotypes, and may be challenging to treat with complete laser coverage in clinically unstable infants, anti-VEGF therapy is often preferred, or used as initial therapy, in these situations within the local protocol [2,26,40]. Post-laser follow-up should include close monitoring for early complications (e.g., hemorrhage and tractional changes) and for treatment gaps requiring additional laser, as outlined in Table 2 and safety considerations [7].

We strongly recommend obtaining retinal imaging before treatment, as late screening or referral may place some infants at imminent risk of retinal detachment. Imaging can be performed using RetCam® or 3nethra Neo®, both available in the UAE [60]. Wide-field retinal cameras (e.g., Optos™) may also support assessment of peripheral changes, when feasible [61]. In addition to treatment planning, imaging facilitates longitudinal documentation to monitor regression, detect recurrence or reactivation, and identify complications over time, and may support remote review workflows where implemented [47,60].

Safety considerations are important. Inappropriately high energy levels or an unfocused laser beam can affect the lens or iris, leading to pupillary constriction or cataract formation [61]. Prolonged duration with shortened intervals can cause excessive energy delivery (“retinal painting”), increasing the risk of collateral tissue effects [62]. During treatment, periodic alignment with the optic nerve and macula is essential, and a safe distance from the macula/fovea should be maintained to reduce the risk of central injury [61]. After laser treatment, caution is warranted before using anti-VEGF agents in eyes with significant fibrovascular proliferation, as rapid changes in vascular activity may be associated with tractional progression in susceptible cases; when sequential therapy is required (e.g., anti-VEGF followed by laser for PAR or reactivation), timing should be guided by disease activity and close follow-up [63,64].

ROP recurrence can occur following anti-VEGF therapy. In such cases, laser treatment is advised six to eight weeks post-injection [64]. Early recurrence signs may include reappearance of plus disease, neovascular endpoints (NVEs), or renewed ridge formation (including “popcorn” lesions) [63]. In the presence of PAR - particularly when exceeding two disc diameters - laser treatment is strongly recommended to reduce the risk of later complications, such as hemorrhage, tears, and detachment [63]. Identifying the demarcation between vascular and avascular retina can be challenging after anti-VEGF; using a 20-D lens for posterior orientation and demarcation, followed by a 28-D or 30-D lens for peripheral assessment, may improve visualization relevant to zonal assessment and treatment planning [65].

Anti-VEGF therapy for ROP: VEGF plays a central role in the pathogenesis of ROP; therefore, intravitreal anti-VEGF agents have emerged as effective therapies by inhibiting aberrant retinal neovascularization. Several agents have been studied in ROP, including bevacizumab, ranibizumab, and aflibercept. Bevacizumab was the most widely used agent initially, largely following the BEAT-ROP trial, which demonstrated superiority over laser therapy in Zone I Stage 3+ disease [66]. Subsequently, the CARE-ROP and RAINBOW studies established the efficacy of ranibizumab, compared with laser therapy [67,68].

Although laser photocoagulation remains a primary treatment modality for many ROP cases, the off-label use of anti-VEGF therapy has increased, particularly in posterior disease (Zone I) and A-ROP, where disease progression may be rapid and complete laser coverage may be challenging. Compared with bevacizumab, ranibizumab has a shorter systemic half-life and lower systemic VEGF suppression, which may reduce potential systemic exposure, albeit with a potentially higher need for retreatment due to later reactivation [64]. In the UAE, ranibizumab is most used, reflecting institutional practice, availability of prefilled syringes, and perceived systemic safety considerations.

We recommend anti-VEGF therapy for A-ROP, Zone I disease at any stage, and Zone II Stage 2-3 disease with plus, consistent with ICROP-III zonal classification and treatment-requiring disease patterns. In cases of severe posterior disease, where vascular landmarks are poorly defined and accurate staging is not feasible, anti-VEGF therapy is also recommended.

For ranibizumab, due to its pre-filled syringe format, it is advised to discard the drug content to the midpoint between the pre-marked line and syringe edge prior to injection [64]. Bevacizumab should be prepared by an in-house pharmacy using sterile technique, appropriately labeled, and stored according to institutional protocols to minimize contamination risk [69]. A 1-cc syringe with a 30-gauge luer-lock needle is recommended for administration.

Intravitreal injections should be performed using separate sterile surgical sets for each eye, with povidone-iodine preparation of the ocular adnexa and injection at 1.7-2.0 mm posterior to the limbus [70]. Procedures are ideally conducted in a negative-pressure room or at the bedside for critically ill infants. Each eye should be treated as a separate procedure, with a nurse and respiratory therapist present. Analgesia (e.g., morphine or ketamine) should be individualized based on GA, systemic condition, and NICU guidance (Table 2).

The ethical considerations and informed consent for off-label anti-VEGF therapy. Anti-VEGF therapy in ROP is off-label. Parents or legal guardians must be informed of the rationale, potential ocular and systemic risks, need for prolonged follow-up, and alternative treatments. Informed consent should be clearly documented, emphasizing the possibility of late reactivation and the uncertainty surrounding long-term systemic and neurodevelopmental effects.

Management of recurrence and reactivation of ROP: Failure of disease resolution, recurrence, or reactivation may occur after both laser and anti-VEGF therapy, with higher reported rates following anti-VEGF treatment [71]. Recurrence requiring retreatment has been reported in 31%-35.7% of infants treated with anti-VEGF agents [71,72].

In accordance with ICROP-III terminology, recurrence refers to the reappearance or progression of active disease after incomplete initial regression, whereas reactivation denotes the return of acute-phase features following a period of apparent regression. Reactivation may occur weeks to months after anti-VEGF therapy, with reported timing ranging from 3 to 16 weeks, and, in some cases, extending beyond this window [73,74].

Indirect retinal laser photocoagulation is the preferred treatment for recurrence or reactivation when feasible. Anti-VEGF therapy may be considered if laser treatment is not possible, or if rapid regression is required due to systemic instability [73]. After laser therapy, re-treatment may be required within two to three weeks if no regression is observed [71]. Given the wide recurrence window after anti-VEGF therapy, we recommend frequent early follow-up (weekly initially) and continued surveillance until at least 60 weeks postmenstrual age, particularly for posterior disease.

Vitreoretinal surgery in ROP: Surgical intervention is generally reserved for Stage 4 and Stage 5 ROP, as defined by ICROP-III. Stage 4 is subdivided into Stage 4A (extrafoveal tractional retinal detachment) and Stage 4B (foveal involvement). Previous reports indicate that progression to total retinal detachment (Stage 5) occurs in approximately 12% of cases, with a median onset of 34 days after anti-VEGF therapy, or six weeks after laser treatment [75,76].

Comprehensive preoperative evaluation of both anterior and posterior segments is essential [77]. While scleral buckle procedures were historically used, lens-sparing vitrectomy (LSV) is now preferred, particularly for Stage 4A disease, due to higher anatomical success rates (up to 90%) and improved potential for functional vision preservation [78-80]. Scleral buckle may still be considered in selected cases, such as non-rhegmatogenous or peripheral detachments. Stage 5 disease typically requires lensectomy, vitrectomy, and membrane peeling, although visual prognosis remains guarded [81].

Adjuvant therapies, including plasmin or preoperative anti-VEGF injections, have been proposed to facilitate surgery; however, their use should be individualized, given limited evidence, and potential systemic risks [82].

Treatment-related complications: Laser photocoagulation is generally safe, but may be associated with short-term systemic effects, such as bradycardia and respiratory stress during or shortly after the procedure, as well as ocular complications ranging from transient refractive changes to more severe outcomes, such as retinal detachment [83]. Long-term sequelae may include high myopia and peripheral visual field loss. Ensuring complete coverage of the avascular retina, confirmed by pre- and post-procedure imaging, reduces the risk of persistent disease, recurrence, and the need for repeat interventions [61].

Anti-VEGF therapy carries risks of short-term ocular complications, including lens injury, vitreous hemorrhage, and vascular occlusion, as well as longer-term concerns, such as myopia, progression of tractional retinal detachment, and delayed or late reactivation of disease [84]. Importantly, systemic VEGF suppression has raised concerns regarding potential effects on organ development and neurodevelopment, although definitive long-term data remain limited [84]. Late recurrence after anti-VEGF therapy, particularly in posterior ROP, is a clinically important consideration and necessitates prolonged and structured follow-up beyond the period typically required after laser treatment. Careful dosing, meticulous technique, and extended surveillance are therefore essential, especially in extremely preterm infants.

Regression, Reactivation, and Long-Term Sequelae of ROP

ROP evolution includes two distinct phases: regression, characterized by disease involution, and reactivation, marked by renewed acute-phase activity [40]. Regression typically occurs more rapidly after anti-VEGF therapy (within one to three days) than after laser treatment or spontaneous resolution, reflecting direct VEGF suppression and altered retinal vascular dynamics. Features include reduced plus disease, peripheral vascularization, whitening of neovascular tissue, and resolution of hemorrhage [40].

Reactivation is more common after anti-VEGF therapy, often occurring between 37 and 60 weeks postmenstrual age, and may follow either complete or partial regression. Manifestations range from new demarcation lines to recurrent Stage 3 disease with plus or progressive fibrosis, leading to detachment [40].

PAR is a clinically important finding, as it increases the risk of late reactivation, hemorrhage, and retinal detachment. Infants with PAR require either laser ablation of avascular areas or close, long-term surveillance. Long-term sequelae of prematurity and ROP include late retinal detachment, macular abnormalities, retinal thinning, vascular anomalies, and secondary glaucoma. These complications may occur years later and underscore the importance of structured, long-term ophthalmic follow-up, even in infants with regressed or mild ROP [40]. *Recommendations for ROP Screening and Anti-VEGF Injection in NICU*

Primary prevention in NICU: Primary prevention of ROP in the NICU is fundamental to reducing the risk of visual morbidity and long-term ophthalmic sequelae in preterm infants [85]. Preventive strategies can be categorized into maternal and perinatal interventions, delivery room and resuscitation practices, and postnatal NICU care, with varying levels of supporting evidence.

Maternal and perinatal interventions include efforts to reduce extreme prematurity and the administration of antenatal corticosteroids, which have strong evidence for improving neonatal outcomes and reducing the incidence and severity of ROP [86]. Delayed cord clamping for 30-60 seconds in preterm infants has also been shown to enhance circulatory stability and reduce complications associated with prematurity, including ROP [87].

Delivery room and neonatal resuscitation strategies emphasize careful oxygen administration. During initial resuscitation, oxygen should be judiciously titrated to avoid hyperoxia, which is a well-established risk factor for abnormal retinal vascular development [88]. Continuous pulse oximetry and gradual adjustment of inspired oxygen concentrations are recommended to minimize oxidative stress in the immature retina.

Postnatal NICU care remains central to ROP prevention. Targeted oxygen saturation management during ongoing NICU care represents one of the strongest evidence-based interventions for ROP risk reduction. Maintaining oxygen saturation within recommended ranges - commonly between 90% and 95% for preterm infants - helps balance the risks of hypoxia-related mortality and hyperoxia-induced retinal injury [85]. Additional preventive measures include strict temperature control [89], caffeine therapy for apnea of prematurity [90], prevention and early treatment of neonatal sepsis, promotion of breast-milk feeding, and rigorous monitoring and regulation of supplemental oxygen exposure [85].

Close monitoring of postnatal growth is also essential, as poor weight gain and growth failure are associated with lower circulating IGF-1 levels, which play a critical role in normal retinal vascularization [91]. Reduced IGF-1 levels contribute to delayed physiological retinal vessel growth and increase susceptibility to pathologic neovascularization, underscoring the importance of adequate nutrition and growth surveillance as part of ROP prevention strategies.

Collectively, these interventions form a comprehensive, evidence-based approach to primary ROP prevention and support optimal visual outcomes in high-risk preterm neonates.

Indication for screening: For ROP screening programs to be efficient and effective, the establishment of a dedicated ROP team within the NICU is essential. This multidisciplinary team should ideally operate under the leadership of a lead neonatologist and include a pediatric ophthalmologist, supported by trained NICU nurses and administrative staff. The neonatologist is primarily responsible for identifying eligible infants and coordinating care, while the pediatric ophthalmologist performs retinal examinations, determines disease severity, and recommends follow-up or treatment. NICU nursing staff play a critical role in tracking screening schedules, documenting examination dates, preparing infants for examinations, and facilitating communication with families. Administrative support is essential to ensure appointment scheduling, documentation completeness, and coordination during transfers or discharge. Comprehensive and standardized documentation is a key component of this process [53].

Screening decisions should be based on the criteria outlined in Table 1. Infants born before 25 weeks of GA should undergo earlier initial screening, particularly when severe comorbidities are present, given their heightened risk for severe ROP [92]. An organized system for the timely identification of infants meeting screening criteria is therefore necessary. Such systems may include nursing-led documentation of anticipated ophthalmic examination dates upon NICU admission, automated alerts within the medical record, and clearly defined responsibility for follow-up scheduling [93].

When infant transfer between facilities is required, all documentation related to ROP screening status, findings, and future screening or treatment requirements should be meticulously recorded and communicated to ensure uninterrupted care. The use of standardized screening forms, and integration within electronic medical record systems, can further support continuity of care and reduce the risk of missed or delayed examinations during transitions [49].

In summary, effective ROP screening in the NICU relies on a clearly defined multidisciplinary team structure, early identification of high-risk infants, precise timing of initial and follow-up examinations, and robust documentation systems to ensure continuity of care across hospital settings.

Intravitreal injections and post-care protocols: Intravitreal anti-VEGF injections in the NICU should be performed by an experienced ophthalmologist, with strict adherence to sterile technique. Treatment is typically administered within 24-48 hours of the treatment decision, to minimize the risk of rapid disease progression while ensuring adequate procedural preparation and safety [49]. Informed parental consent is mandatory, particularly given the off-label use of anti-VEGF agents in neonates, and should include discussion of potential ocular and systemic risks and benefits.

Each eye must be treated as a separate procedure, using independent sterile instruments and preparation, to prevent cross-contamination. Infants should be kept nil by mouth (NPO) prior to the procedure, with fasting duration individualized based on GA, weight, and local sedation or anesthesia protocols. Continuous monitoring and appropriate PPE are essential throughout the procedure.

Post-procedure care includes maintenance of intravenous fluids and enteral feeding as prescribed, and close monitoring for eyelid edema, infection, intraocular bleeding, or cardiorespiratory instability. Topical moxifloxacin eye drops every six hours for one week are recommended, with vigilance for potential adverse reactions and modification if contraindicated. Vital signs, including blood pressure, heart rate, and oxygen saturation, should be monitored closely in the immediate post-injection period [49].

A follow-up ophthalmic examination is recommended within 48-72 hours after injection, with subsequent assessments based on disease severity. Urgent ophthalmic review is warranted if signs of infection, increased intraocular pressure, worsening plus disease, or retinal hemorrhage are suspected. All post-procedure findings, instructions, and follow-up plans should be clearly documented [49].

Unmet Medical Needs in ROP Management

Despite advances in neonatal and ophthalmic care, several unmet needs persist in the management of ROP. Standardized documentation of retinal findings remains suboptimal in many regions, underscoring the need for universal adoption of ICROP-III terminology to enhance clinical consistency, research comparability, and telemedicine implementation. Variability in treatment indications and follow-up protocols continues to exist, and current recommendations for Zone I ROP remain largely based on the ETROP trial, highlighting the need to update local protocols in line with emerging evidence [94].

Limited awareness of optimal oxygen management and primary prevention strategies among neonatologists and NICU nurses represents a critical gap. This emphasizes the need for structured education programs, ongoing training, and competency assessments for NICU teams to strengthen ROP prevention efforts [95,96].

As the population of infants at risk for ROP continues to grow, particularly in low- and middle-income settings, reliance solely on expert ophthalmologists for screening poses challenges. Expanding access through telemedicine, wide-field retinal imaging, and trained mid-level healthcare personnel may help improve screening coverage and reduce delays in diagnosis [94]. Strengthening neonatal care, improving access to quality screening and treatment services, engaging office staff in post-discharge follow-up, and ensuring coordinated care are essential strategies to mitigate visual impairment from ROP [49,97].

Effective communication among NICU staff, ophthalmologists, and families is vital to ensure timely screening and follow-up. Clearly defined written protocols, standardized discharge summaries, and explicit assignment of responsibilities are particularly important during inter-hospital transfers and after NICU discharge, to prevent gaps in care [47,92,96,98].

Finally, the development of integrated care pathways, supported by electronic tracking systems, represents a forward-looking approach to improving continuity, coordination, and quality of ROP care across healthcare settings, reducing the risk of missed screenings and delayed interventions [99].

## Conclusions

In this national consensus, we present unified recommendations for the classification, screening, and management of ROP in the UAE. These recommendations aim to standardize clinical practice, promote early detection, guide timely intervention, and improve coordination of care across neonatal and ophthalmic services. We also address the management of recurrent or reactivated disease and outline the role of surgical interventions in advanced stages of ROP. By identifying key unmet needs - including variability in practice, gaps in neonatologist engagement, and challenges in long-term follow-up - this consensus underscores the importance of multidisciplinary collaboration and system-level solutions.

Implementation of these recommendations, supported by integrated care pathways, structured NICU staff training, and robust follow-up frameworks, has the potential to improve consistency of care and optimize long-term visual outcomes for preterm infants. Future efforts should focus on translating these recommendations into practice through education, audit, and the development of standardized clinical algorithms to support sustainable, high-quality ROP care nationwide.

## Appendices

Appendix A

**Table 3:** Pre-meeting Questionnaire: Management of Retinopathy of Prematurity

Domain	Question	Response Type
A. Participant Profile	1. Professional Background (Pediatric Ophthalmologist / Retina Specialist / Neonatologist / Other)	Multiple Choice
	2. Years of Experience Managing ROP	Numeric (Years)
	3. Type of Institution (Government / Private / University / NGO)	Multiple Choice
	4. Approximate Number of ROP Infants Managed per Month	Numeric
B. Screening Practice	5. What Criteria Are Used for Screening (Gestational Age, Birth Weight, or Additional Risk Factors)?	Open-Ended
	6. Who Performs ROP Screening in Your Center?	Multiple Choice
	7. What Screening Method Is Used? (Indirect Ophthalmoscopy / Digital Imaging / Both)	Multiple Choice
	8. At What Postnatal Age Is The First Screening Typically Performed?	Numeric (Weeks)
	9. What Interval Is Used for Follow-Up Screening?	Numeric / Open-Ended
C. Documentation and Grading	10. Do You Use a Standardized ROP Documentation Form or Digital Record?	Yes / No
	11. Do You Follow the International Classification (ICROP3)?	Yes / No
	12. Are Imaging Systems (RetCam or Smartphone-Based) Routinely Available?	Yes / No
D. Treatment Practice	13. What Is the Preferred Treatment for Type 1 ROP? (Laser / Anti-VEGF / Combination)	Multiple Choice
	14. Who Performs ROP Treatment in Your Institution?	Multiple Choice
	15. What Is the Typical Time Interval Between Diagnosis and Treatment?	Numeric (Days)
	16. How Do You Follow Up After Treatment? (Schedule and Method)	Open-Ended
E. Referral, Networking, and Tele-ROP	17. Are ROP Referrals Between NICUs and Ophthalmologists Structured or Ad Hoc?	Structured / Ad-hoc
	18. Is Tele-ROP (Digital Imaging or Remote Interpretation) Available or Under Development in Your Area?	Yes / No
	19. If Yes, Which Platform or System Is Used?	Open-Ended
F. Training and System Gaps	20. Are There Regular Training Opportunities for Ophthalmology Residents or Fellows?	Yes / No
	21. What Are the Major Barriers to Optimal ROP Management in Your Region?	Open-Ended
G. Consensus Priorities	22. Rank the Following Areas Where National Guidelines Are Most Needed: Screening Criteria, Timing, Treatment Thresholds, Anti-VEGF Use, Follow-Up Schedule, Referral Systems	Ranking (1-5)
	23. Additional Comments or Suggestions Before the Meeting	Open-Ended
